# The ICD-11 Diagnoses in the Mental Health Field – An Innovative Mixture

**DOI:** 10.32872/cpe.10647

**Published:** 2022-12-15

**Authors:** Andreas Maercker

**Affiliations:** 1 Department of Psychology, Division of Psychopathology and Clinical Intervention, University of Zurich, Zurich, Switzerland

The development of ICD-11 in the mental health field has been innovative in several ways. Perhaps most notable is that it has become equally relevant to clinicians and researchers. Before discussing these two aspects in more detail, it should be mentioned that the processes by which the ICD-11 was created were also innovative and, moreover, that clinical psychologists and psychiatrists were equally involved at several crucial points in the ICD-11 development. This began with Dr. Geoffrey Reed, a US clinical and medical psychologist, as the responsible WHO senior project officer for new developments in the mental health field and who set important impulses at all stages of the process (e.g., Reed, 2010).

From the beginning, the Lebanese psychologist Brigitte Khoury and the Mexican psychologist Maria Elena Medina-Mora served on the International Advisory Group for this field. Both have published on important milestones and outcomes of regional meetings (Khoury et al., 2011; Medina-Mora et al., 2019). Furthermore, the author of this editorial, in his capacity as a psychologist, was one of the working group leaders of the ICD-11 development (Maercker et al., 2013). This new way of composing decision-making bodies represented an important step in the development of the international Mental and Behavioral Disorder classification. This was further supported by the inclusion of clinicians and researchers from the fields of clinical social work and psychiatric nursing sciences in the committees. Thus, the whole ICD-11 development relied on a very multidisciplinary process.

What, then, were the innovations for clinicians worldwide? From the very start, the aim was that “clinical usability” should be the focus of development (First et al., 2015). The rationale for this was that global applicability should be ensured both in countries with few and with ample health system resources. The intention was to avoid creating complex and costly diagnostic algorithms that would be unrealistic for the time and human resources available in some regions of the world. Regarding clinical usability, the arguments were also based on the limited memory capacity for information elements known from general psychology, which typically does not allow for an overly complex diagnostic decision process without the loss of information. Here, experts distinguished their approach from highly complex diagnostic algorithms in the DSM (Diagnostic and Statistical Manual of Mental Disorders), which, for example, had different minimum numbers of required symptoms for several symptom groups. In addition, the DSM in its various versions contained lists of symptoms and criteria that grew longer and were almost unmanageable in each new version (DSM-III, DSM-III-R, DSM-IV, DSM-IV-TR).

Therefore, the International Advisory Group made a preliminary decision to follow a prototype approach to disorder definitions. This meant that a few symptoms define the core of a diagnosis (core symptoms or essential features), with a number of other associated symptoms (accessory symptoms or additional clinical features), which must not all be present to assign a diagnosis. The International Advisory Group also made the decision to omit subtypes from the diagnoses as much as possible, which was later widely adopted in the ICD-11 development.

Further means of increasing clinical usability was the introduction of new sections in the definition texts: e.g., Boundary with Normality, Developmental Presentations, Culture-Related Features, Sex- and/or Gender-Related Features, Boundaries with Other Disorders and Conditions (Differential Diagnosis). These helpful new sections of ICD-11 are discussed in most of the articles in this Special Issue. These sections are, in fact, included as standard in the central internet publication of ICD-11 as so-called *Clinical Descriptions and Diagnostic Recommendations* (CDDR) and, as with all material from the WHO, are also available free of charge.

How about the scientific innovations? It is impossible to list all innovations in the present context. In terms of methodology, innovations were based on the serious consideration of and alignment with the customer orientation. Customers of a classification system include the global clinicians or practitioners, as well as the patients or clients in the health care system – Both of these groups were involved throughout the entire process. Furthermore, survey studies were conducted with the World Associations of Psychologists and Psychiatrists to ask about previous diagnostic habits, as well as missing, problematic, and stigmatizing diagnoses (Robles et al., 2014). The results of these studies were implemented whenever possible. For example, 12% of these studies (of over 3200 clinicians from 13 countries across six continents) indicated a need for a diagnosis that went beyond "classic" PTSD to include more complex trauma sequelae. This finding informed the development of the diagnosis of complex PTSD that now exists in ICD-11 (see the paper in this Special Issue). Moreover, the patients or people affected by the disorders were also involved in the feedback process of the ICD-11 development (Hackmann et al., 2019).

For the subsequent steps of ICD-11 finalization, the Global Clinical Practice Network (https://GCP.network) handled the involvement of global clinicians and practitioners. This network operates in nine world languages (including six European languages) and comprises approximately 10,000 people to date (operating in collaboration with Columbia University, New York). Beta versions of the new diagnostic proposals were submitted to this network in 2015, and for more recent surveys, the revised diagnoses were also submitted for further review. It is noteworthy to mention that one can also enroll in online continuing education courses in this network.

It is impossible to provide an overview of the various innovations and their details here, as they are too extensive for an overview. This Special Edition of *Clinical Psychology in Europe (CPE)* is very pleased to present five very different topic areas: The Autism Spectrum Disorder (which belongs to the Neurodevelopmental Disorders), the Disorders Specifically Associated with Stress (a separate subchapter), the Personality Disorders (also a separate subchapter), the Disorders of Substance Use (with the emphasis here on Alcohol Use and a smaller focus on Addictive Behaviors), as well as Chronic Pain (a separate, overarching subchapter).

It is very fortunate that our journal *Clinical Psychology in Europe* is addressing the topic of ICD-11 diagnoses, and as mentioned earlier, that many other regions of the world have already highlighted it as an area of particular prominence and innovation. It is interesting to note that the majority of international research activities on the individual disorders of ICD-11 come from outside the United States, with European research activities playing a prominent role. Not incidentally, these activities merge closely with WHO-sponsored programs on culturally appropriate interventions for global application (Heim & Kohrt, 2019; Heim et al., 2021). However, in recent years, there has also been an incipient trend of an increasing number of US studies being devoted to ICD-11 (e.g., Cloitre et al., 2019). CPE will certainly continue to have a focus on contributions related to this global classification system, which is equally useful for both clinicians and researchers.

## References

[r1] Cloitre, M., Hyland, P., Bisson, J. I., Brewin, C. R., Roberts, N. P., Karatzias, T., & Shevlin, M. (2019). ICD‐11 posttraumatic stress disorder and complex posttraumatic stress disorder in the United States: A population‐based study. Journal of Traumatic Stress, 32(6), 833–842. 10.1002/jts.22454 31800131

[r2] First, M. B., Reed, G. M., Hyman, S. E., & Saxena, S. (2015). The development of the ICD‐11 clinical descriptions and diagnostic guidelines for mental and behavioural disorders. World Psychiatry: Official Journal of the World Psychiatric Association (WPA), 14(1), 82–90. 10.1002/wps.20189 25655162 PMC4329901

[r3] Hackmann, C., Balhara, Y. P. S., Clayman, K., Nemec, P. B., Notley, C., Pike, K., Reed, G. M., Sharan, P., Rana, M. S., Silver, J., Swarbrick, M., Wilson, J., Zeilig, H., & Shakespeare, T. (2019). Perspectives on ICD-11 to understand and improve mental health diagnosis using expertise by experience (INCLUDE Study): An international qualitative study. The Lancet Psychiatry, 6(9), 778–785. 10.1016/S2215-0366(19)30093-8 31296444

[r4] Heim, E., & Kohrt, B. A. (2019). Cultural adaptation of scalable psychological interventions: A new conceptual framework. Clinical Psychology in Europe, 1(4), e37679. 10.32872/cpe.v1i4.37679

[r5] Heim, E., Mewes, R., Abi Ramia, J., Glaesmer, H., Hall, B., Harper Shehadeh, M., Ünlü, B., Kananian, S., Kohrt, B. A., Lechner-Meichsner, F., Lotzin, A., Moro, M. R., Radjack, R., Salamanca-Sanabria, A., Singla, D. R., Starck, A., Sturm, G., Tol, W., Weise, C., & Knaevelsrud, C. (2021). Reporting Cultural Adaptation in Psychological Trials – The RECAPT criteria. Clinical Psychology in Europe, 3(Special Issue), e6351. 10.32872/cpe.6351 36405678 PMC9670826

[r6] Khoury, B., Loza, N., & Reed, G. M. (2011). Arab specificities, Arab voice and global connectedness: The development of WHO’s new international classification of mental disorders (ICD11). Arab Journal of Psychiatry, 22(2), 95–99.

[r7] Maercker, A., Brewin, C. R., Bryant, R. A., Cloitre, M., van Ommeren, M., Jones, L. M., Humayan, A., Kagee, A., Llosa, A. E., Rousseau, C., Somasundaram, D. J., Souza, R., Suzuki, Y., Weissbecker, I., Wessely, S. C., First, M. B., & Reed, G. M. (2013). Diagnosis and classification of disorders specifically associated with stress: Proposals for ICD‐11. World Psychiatry: Official Journal of the World Psychiatric Association (WPA), 12(3), 198–206. 10.1002/wps.20057 24096776 PMC3799241

[r8] Medina-Mora, M. E., Robles, R., Rebello, T. J., Domínguez, T., Martínez, N., Juárez, F., Sharan, P., & Reed, G. M. (2019). ICD-11 guidelines for psychotic, mood, anxiety and stress-related disorders in Mexico: Clinical utility and reliability. International Journal of Clinical and Health Psychology, 19(1), 1–11. 10.1016/j.ijchp.2018.09.003 30619492 PMC6300716

[r9] Reed, G. M. (2010). Toward ICD-11: Improving the clinical utility of WHO’s International Classification of mental disorders. Professional Psychology, Research and Practice, 41(6), 457–464. 10.1037/a0021701

[r10] Robles, R., Fresán, A., Evans, S. C., Lovell, A. M., Medina-Mora, M. E., Maj, M., & Reed, G. M. (2014). Problematic, absent and stigmatizing diagnoses in current mental disorders classifications: Results from the WHO-WPA and WHO-IUPsyS Global Surveys. International Journal of Clinical and Health Psychology, 14(3), 165–177. 10.1016/j.ijchp.2014.03.003

